# HIV infection drives proinflammatory adipocyte differentiation in an *in vitro* model and reveals a new inflammatory pathway

**DOI:** 10.3389/fcimb.2025.1627963

**Published:** 2025-07-17

**Authors:** Rosa Nicole Freiberger, Cynthia Alicia Marcela López, Franco Agustin Sviercz, Patricio Jarmoluk, María Belén Palma, Marcela Nilda García, Jorge Quarleri, M. Victoria Delpino

**Affiliations:** ^1^ Instituto de Investigaciones Biomédicas en Retrovirus y Sida (INBIRS), Facultad de Medicina, Consejo de Investigaciones Científicas y Técnicas (CONICET), Universidad de Buenos Aires, Buenos Aires, Argentina; ^2^ Cátedra de Citología, Histología y Embriología, Facultad de Ciencias Médicas, Universidad Nacional de La Plata, Buenos Aires, Argentina; ^3^ Instituto de Neurociencias (INEU-CONICET), Laboratorio de Investigación Aplicada en Neurociencias (LIAN), Fundación para la Lucha contra las Enfermedades Neurológicas de la Infancia (FLENI), Escobar, Buenos Aires, Argentina

**Keywords:** HIV, adipocyte, MSCs, lipid droplets (LD), adipogenesis

## Abstract

**Introduction:**

Adipose tissue regulates metabolic homeostasis and serves as a reservoir for mesenchymal stem cells (MSCs), which differentiate into osteoblasts and adipocytes, balancing bone and lipid metabolism. Bone loss and fat accumulation are common in individuals living with HIV, prompting us to investigate how R5- and X4-tropic HIV modulates adipocyte differentiation and tissue homeostasis using an in vitro model of MSC-derived adipogenesis.

**Methods:**

The study used an *in vitro* model of MSCs to examine how R5- and X4-tropic HIV strains affect adipocyte differentiation and function. Researchers assessed adipogenesis by analyzing lipid droplet formation, expression of adipogenic transcription factors (C/EBPα, C/EBPβ, PPAR-γ), lipogenic/lipolytic enzymes, SREBPs, cytokine secretion, and the effects of CXCR4 and CCR5 with specific inhibitors.

**Results:**

HIV exposure influences adipogenesis, increasing lipid droplet size in a tropism dependent manner and upregulating key adipogenic factors such as C/EBPα, C/ EBPβ, and PPAR-γ. This process involves the regulation of lipogenic and lipolytic enzymes, lipid droplet-lysosome interactions, and potential lipid droplet mitochondria cross-talk to fuel lipid accumulation. Additionally, HIV modulates sterol regulatory element-binding proteins (SREBPs), which control fatty acid, triacylglycerol, and cholesterol synthesis. Notably, SREBP2 downregulation correlates with increased type I interferons (IFNa2, IFNb1), linking lipid metabolism to immune responses in HIV infection. HIV-infected adipocytes also exhibit an increased leptin/adiponectin ratio and enhanced IL-1b and IL-6 secretion, contributing to the inflammatory state observed in people with HIV. CXCR4 plays a key role in adipocyte differentiation, as its inhibition with AMD3100 reduces adipocyte number, size, and lipid droplet accumulation under X4-tropic HIV exposure. In contrast, CCR5 does not appear to be significantly involved in adipose tissue homeostasis under R5-tropic HIV exposure.

**Discussion:**

These findings, derived from an *in vitro* model, suggest that HIV alters MSC differentiation into adipocytes, impacting adipose tissue homeostasis and function.

## Introduction

Previous studies have shown that a pathological decline in bone mineral density is often accompanied by an increase in fat accumulation in bone tissue (Meunier et al., 1971; Verma et al., 2002; Yeung et al., 2005). Even with highly active antiretroviral therapy (HAART), many individuals living with HIV-1 experience metabolic abnormalities, including lipodystrophy, dyslipidemia, and hematological changes, alongside reduced bone mineral density (Mccomsey et al., 2010; Feeney and Mallon, 2011; Durandt et al., 2019). Initially attributed to antiretroviral therapy (ART) toxicity, the persistence of these abnormalities in ART-naïve HIV-infected individuals suggests that the virus itself plays a direct role in their development (Bruera et al., 2003; Koethe et al., 2020).

Adipogenesis is regulated by key transcription factors, including peroxisome proliferator-activated receptor gamma (PPAR-γ) and CCAAT enhancer-binding protein alpha (C/EBPα) (Rosen and Macdougald, 2006). Interestingly, PPAR-γ drives adipocyte differentiation and suppresses osteoblastogenesis by inhibiting Runt-related transcription factor 2 (Runx2), a master regulator of osteoblast commitment (Jeon et al., 2003). This interplay highlights the complex balance between these lineages and the potential for their disruption in pathological conditions (Bennett et al., 1991; Beresford et al., 1992; Muruganandan et al., 2009). Adipocytes and osteoblasts also share several genetic, hormonal, and environmental regulatory factors, and they exhibit a remarkable degree of plasticity, allowing for interconversion under certain conditions. Furthermore, increased adipocyte accumulation in bone marrow can disrupt bone remodeling by altering osteoblast function and regulating the development and activity of osteoclasts, which contribute to bone resorption (Muruganandan et al., 2020).

In response to hypoxia, cellular stress, and hypertrophy, adipocytes produce various cytokines and adipokines (e.g., TNF-α, IL-6, leptin, and adiponectin) that regulate immune responses and inflammation (Trayhurn and Wood, 2004).

Although productive infection of mesenchymal stem cells (MSCs) has not been conclusively demonstrated, these cells are believed to be susceptible to HIV infection (Couturier et al., 2015; Damouche et al., 2015). Adipose tissue has been proposed as a potential reservoir for HIV-1, as adipocytes express CD4, CCR5, and CXCR4 receptors necessary for viral entry (Hazan et al., 2002; Nazari-Shafti et al., 2011). *In vitro* studies have shown that adipocytes can harbor HIV-1 (Maurin et al., 2005). However, the presence of HIV-1 within adipocytes has not been conclusively reported (Dupin et al., 2002; Munier et al., 2003). Beyond its role in metabolic regulation, adipose tissue contains MSCs that are critical for maintaining lipid homeostasis, with the ability to differentiate into adipocytes (Hu et al., 2018; Souza-Moreira et al., 2019).

In this study, we examine whether HIV exposure influences MSC differentiation into adipocytes and how this affects lipid metabolism and adipose tissue homeostasis. By elucidating the mechanisms underlying adipose tissue dysfunction in people living with HIV, our findings provide insight into the metabolic and inflammatory complications associated with the virus.

## Materials and methods

### Isolation and expansion of MSCs

The umbilical cords were preserved in α-Minimal Essential Medium (α-MEM, Gibco) and handled following previously outlined procedures (Palma et al., 2021).

In summary, each umbilical cord was cut into 5 mm fragments, with a sagittal incision made to expose Wharton’s jelly. Careful removal of umbilical blood vessels was performed using clamps. The fragments were then washed 2 or 3 times with Dulbecco’s phosphate-buffered saline from Sigma-Aldrich to eliminate residual blood. The section with the exposed jelly was placed face down at the bottom of a culture plate, and α-MEM supplemented with 10% platelet lysate was added. The plates were then incubated at 37°C in a humid atmosphere containing 5% CO2, with the cell culture medium replenished every 2 to 3 days. Expansion of umbilical cord-derived MSCs was typically observed between 10 to 14 days after explanation, and cells were further amplified until reaching passage 2/3. MSCs were characterized by the expression of CD105, CD73, and CD90, and the absence of CD45, CD34, CD14, CD19, and HLA-DR molecules (Dominici et al., 2006). For experiments, MSCs were cultured in α-MEM supplemented with 10% heat-inactivated fetal bovine serum (Gibco-BRL, Life Technologies, Grand Island, NY), 100 U/ml of penicillin, and 100 mg/ml of streptomycin (complete medium) and were utilized until passage 5. The cells were routinely tested for mycoplasma contamination using the MycoAlert^®^ Mycoplasma detection kit (LT07-318, Lonza, Tampa, FL, USA).

This study received approval from the Comité de Ética de la Facultad de Ciencias Médicas de la Universidad de Buenos Aires, Argentina (RESCD-2023-1291). Written informed consent was obtained from each mother before normal cesarean birth, and human umbilical cords were collected from discarded placentas.

### Cell-free wild-type HIV infection of MSCs and adipocytes

The full-length infectious clones of wild-type (WT)-HIV AD8 and NL43 strains were obtained from the NIH AIDS Reagent Program (Division of AIDS, NIAID, NIH, USA).

The quantification of HIV capsid (p24 antigen) in viral stocks was conducted using a commercial ELISA assay (INNOTEST^®^ HIV Antigen mAb).

### Adipocyte differentiation

MSCs were seeded at a density of 5×10^4^ cells per well in 24-well plates and allowed to reach confluence. Subsequently, the culture medium was replaced with an adipocyte differentiation medium, consisting of DMEM, 0.5 mM 3-isobutyl-1-methylxanthine (IBMX), 0.1 µM dexamethasone, 50 μM indomethacin, and 10 µg/ml human insulin, all obtained from Sigma Aldrich, St. Louis, MO, USA (Crivaro et al., 2020, 2023). Complete differentiation was attained within the timeframe of days 7 to 10.

### Surface expression of CD4, CXCR4, and CCR5 during adipocyte differentiation

At 0, 3, and 7 days after initiating the adipocyte differentiation, the cells were washed and subjected to staining for surface antigens. This staining procedure involved incubation with the following antibodies all used at a 1:100 dilution for 30 minutes at 4°C: FITC Mouse anti-human CD195 (561747) (BD PharmingenTM, United States), PE Mouse Anti-Human CD184 (555974) (BD PharmingenTM, United States), and PerCP anti-human CD4 antibody (344624) (Biolegend, United States). Data were acquired using a Full Spectrum Flow Cytometry Cytek^®^ Northern Lights 3000™ (Cytek Biosciences Inc., USA) and analyzed with FlowJo.v10.6.2 (Ashland, USA).

### HIV-infections using X4- and R5-tropic strains

MSCs and differentiated adipocytes (at 7 days) were infected with an inoculum of 1 pg of p24/cell using the CCR5-tropic HIV strain AD8 and the CXCR4-tropic HIV strain NL43, following the previously described procedure (Freiberger et al., 2024; Lopez et al., 2024). Infection efficiency and replication were evaluated by measuring intracellular p24 expression using the KC57 monoclonal antibody labeled with phycoerythrin against p24 (PE-KC57 [FH190-1-1] (6604667) protein (Beckman Coulter, United States) used at a 1:250 dilution via confocal microscopy using a Zeiss LSM 800 confocal microscope (Zeiss, Jena, Germany).

The detection of HIV proviral DNA was performed using the Alu-PCR technique, following the protocol described by Kumar et al. (2002). MSCs and adipocytes cells exposed to HIV, along with ACH-2 cells (used as a positive control), were centrifuged in 1.5-ml microtubes at 16,000 g for 5 minutes. After carefully removing and discarding the supernatants, cell pellets were resuspended in lysis buffer (10 mM Tris-HCl, pH 8.0, 50 mM KCl, 400 µg/ml proteinase K; Invitrogen) at appropriate concentrations (20x10^6^ cells/ml for ACH-2 cells; 5x10^6^ to 10x10^6^ cells/ml for LX-2) and digested for 12 to 16 hours at 55°C in a heating shaker. Proteinase K was then inactivated by heating the digested samples at 95°C for 5 minutes. The cell lysates were either immediately used for HIV DNA quantification or stored at -70°C until further analysis.

Integrated HIV DNA determination was carried out in a 50 µl reaction mixture containing Taq polymerase buffer (Invitrogen), 3 mM MgCl2, 300 mM deoxynucleoside triphosphates (Invitrogen), 300 nM of each of the 4 primers, and 2.5 U Taq polymerase (Invitrogen).

The primers used for preamplification were:

ULF1: 5´-ATG CCA CGT AAG CGA AAC TCT GGG TCT CTC TDG TTA GAC-3´
Alu1: 5´- TCC CAG CTA CTG GGG AGG CTG AGG-3´
Alu2: 5´- GCC TCC CAA AGT GCT GGG ATT ACA G-3´
For Real-time PCR, the following primers were used:
Lambda T: 5´-ATG CCA CGT AAG CGA AAC T-3´
UR2: 5´-CTG AGG GAT CTC TAG TTA CC-3´
UHIV TaqMan LC640: 5´-CAC TCA AGG CAA GCT TTA TTG AGG C-3´.

### Co-receptor antagonists

We treated MSCs with the CXCR4 antagonist AMD3100 (20 μM), or the CCR5 antagonist TAK-779 (20 μM) (all from Sigma-Aldrich, Argentina). Each inhibitor was applied separately, and the cells were incubated at 37°C for 1 hour. Following this pre-incubation, the cells were exposed to HIV.

### Determination of cell death

The percentage of cellular death was assessed by staining with 7-AAD apoptosis detection kit (BD Biosciences, United States). Staurosporine stimulation was used as a positive control of cell death. Data were acquired using a Full Spectrum Flow Cytometry Cytek^®^ Northern Lights 3000™ (Cytek Biosciences Inc., USA) and analyzed with FlowJo.v10.6.2 (Ashland, USA).

### Assessment of mitochondrial reactive oxygen species generation

mROS, which includes superoxide, was measured using flow cytometry. The cells were stained with 5 μM MitoSOX™ (catalog number M36008; Thermo Fisher Scientific) for 30 minutes. This fluorescence-based assay employs a positively charged probe that swiftly accumulates within mitochondria, where it becomes susceptible to oxidation by ROS. Rotenone stimulation was used as the positive control. Data were acquired using a Full Spectrum Flow Cytometry Cytek^®^ Northern Lights 3000™ (Cytek Biosciences Inc., USA) and analyzed with FlowJo.v10.6.2 (Ashland, USA).

### Cellular mRNA preparation and RT-qPCR

Total cellular mRNA was extracted using Quick-RNA MiniPrep Kit (Zymo Research) and 1 µg of RNA was employed to perform the reverse transcription by means Improm-II Reverse Transcriptase (Promega). Quantitative reverse transcription-polymerase chain reaction (RT-qPCR) analysis was achieved by run on a StepOne real-time PCR detection system (Life Technology) using SYBR Green as fluorescent DNA binding dye. Primers sequences used for amplification were the following: β-actin sense 5- CCTGGCACCCAGCACAAT-3, antisense 5- CGGGATCCACACGGAGTACT-3; PPAR-γ sense 5- GGCCGCAGATTTGAAAGAAG-3, antisense 5- GTTTGAGAAAATGGCCTTGTTGT-3; C-EBPα sense 5- CCAAGAAGTCGGTGGACAAGA-3, antisense 5- ATTGTCACTGGTCAGCTCCA-3; C-EBPβ sense 5- TACTACGAGGCGGACTGCTT-3, antisense 5- CTGGTAGCCGAGGTAAGCG-3; hormone-sensitive lipase (HSL) sense 5- CATCTCCATTGGGCTGGTGT-3, antisense 5- ATCTCAAAGGCTTCGGGTGG-3; lipoprotein lipase (LPL) sense 5- ATCCGCGTGATTGCAGAGAG-3, antisense 5- GATGAATGGAGCGCTCGTGG-3; adipose triglyceride lipase (ATGL) sense 5- CAAGCGGAGGATTACTCGCA-3, antisense 5- CAAGCGGATGGTGAAGGACA-3; diglyceride acyltransferase (DGAT)1 sense 5- CCGGACAATCTGACCTACCG-3, antisense GGGATGTTCCAGTTCTGCCA; DGAT2 sense 5- GCCTGTGTTGAGGGAGTACC-3, antisense 5- CAGGGCCAGTTTCACAAAGC-3; sterol regulatory element-binding proteins (SREBP)1 sense 5- GGGACCACTGTCACTTCCAG-3, antisense 5- TTCAAAGCTTCGACGCAGG-3; SREBP2 sense 5- ATGGGCAGCAGAGTTCCTTC-3, antisense 5- CGACAGTAGCAGGTCACAGG-3; IFNβ1 sense 5- ACGCCGCATTGACCATCTAT-3, antisense 5- GTCTCATTCCAGCCAGTGCT-3; IFNα2 sense 5- CTTGTGCCTGGGAGGTTGTC-3, antisense 5- GGTGAGCTGGCATACGAATCAA-3. Lysosomal acid lipase (LIPA) sense 5 ′-GTGGGTCATTCTCAAGG-CACCA-3′, antisense 5 ′-CCATAGGGCTAGTACAGAAGGC-3′; PPAR-α sense 5 ′-AAGCAAAACT-GAAAGCAGAA-3′, antisense 5 ′GTCTTCTCAGCCATACACAG3 ′

The amplification cycle for PPAR-γ, C-EBPα, C-EBPβ, HSL, LPL, ATGL, DGAT, DGAT2, SREBP1, SREBP2, IFNβ1, IFNα2 was 95°C for 15s, 59°C for 30s and 72°C for 60s. For β-actin, PPAR-α and LIPA was 95 °C for 15 s, 57 °C for 30 s, and 72 °C for 60 s.

All primer sets yielded a single product of the correct size. Relative transcript levels were calculated using the 2^-ΔΔCt^ method using β-actin as a normalizer gene. The Ct values for β-actin showed no significant variation between infected and non-infected samples or across different time points (ΔCt < 0.5), confirming its stability and suitability for normalization.

### Glycerol determination

Glycerol release from differentiated adipocytes was quantified using a modified version of the method of Garland and Randle (Garland and Randle, 1962). Briefly, culture medium was aspirated and cells were washed once with phosphate-buffered saline (PBS). Thereafter, adipocytes were incubated in PBS supplemented with 2% (w/v) fatty-acid-free bovine serum albumin (BSA) for 6 h at 37°C in a humidified 5% CO_2_ atmosphere. Following incubation, aliquots of the extracellular medium were collected and centrifuged at 1,000 × g for 5 min to remove any cellular debris. Glycerol concentration in the cleared supernatant was determined using the TG Color GPO/PAP AA enzymatic assay kit (Wiener, Buenos Aires, Argentina), according to the manufacturer’s protocol. Absorbance was read at 505 nm on a microplate reader.

### Intracellular triglyceride and cholesterol quantification

Differentiated adipocytes were lysed in PBS containing 1% (v/v) Triton X-100, and the resulting lysates were used for both triglyceride and cholesterol determinations. For triglycerides, intracellular lipids were extracted directly in the Triton X-100 buffer and enzymatically hydrolyzed to glycerol and free fatty acids using lipase as specified in the TG Color GPO/PAP AA kit protocol (Wiener, Buenos Aires, Argentina), after which released glycerol was quantified by measuring absorbance at 505 nm on a microplate reader. Total cholesterol was measured in the same lysates using the Colestat enzymatic assay kit (Wiener, Buenos Aires, Argentina) according to the manufacturer’s instructions, with absorbance also read at 505 nm. All lipid values were normalized to total protein content, which was determined by the Bradford assay (Bio-Rad Protein Assay Kit) using bovine serum albumin as the standard.

### Assessment of adipocyte differentiation measuring lipid droplet accumulation

Adipocyte differentiation was examined using Bodipy 493/503 (Life Technologies). Cells cultured in 24-well plates were fixed with 10% formalin for one hour, followed by permeabilization using 0.3% Triton X100. Subsequently, lipid droplets were stained with 1 µg/mL of Bodipy 493/503 from Invitrogen. For nuclear visualization, DAPI (Thermo Scientific) was used for counterstaining. The prepared coverslips were mounted in a PBS-glycerin solution (9:1 v/v) and analyzed using a Zeiss LSM 800 confocal microscope (Zeiss, Jena, Germany) with 10× and 40× objectives. This method involved quantifying five microscopic fields per well, within two wells per condition, for each experimental set.

### Adipocyte morphology

Adipocyte morphology was further evaluated using Differential Interference Contrast (DIC) microscopy, acquired in parallel with the confocal fluorescence images. DIC and fluorescence channels were superposed to enable precise identification of individual adipocytes based on their characteristic round shape and the presence of intracellular lipid droplets. Cell borders were manually delineated using Fiji software (ImageJ, National Institutes of Health, Bethesda, MD, USA), and the cross-sectional area of each adipocyte was measured. Only clearly defined and isolated cells were included in the analysis.

### Lysosome and mitochondria staining

For lysosomal staining, cells were treated with 0.5 mmol/L Lysotracker Red (Life Technologies) in full medium for 45 minutes before fixation. Following this, cells were fixed with 4% paraformaldehyde (PFA) in PBS for 10 minutes at room temperature.

To identify mitochondria, cells were stained with MitoTracker Deep Red (500 nM, Life Technologies) for 30 minutes at 37°C and then fixed as described.

Nuclei were visualized using DAPI from Thermo Scientific for counterstaining. Confocal images were examined using a Zeiss LSM 800 confocal microscope (Zeiss, Jena, Germany). To quantify the degree of colocalization between Bodipy 493/503-labeled LD and the LysoTracker probe, as well as between Bodipy 493/503-labeled LD and the MitoTracker probe, we calculated the Manders’ correlation coefficient from the corresponding confocal images using FIJI software (ImageJ, National Institutes of Health, Bethesda, MD, USA) (Manders et al., 1993).

### Statistical analysis

Where applicable, statistical analysis was performed with one-way ANOVA. Multiple comparisons between all pairs of groups were made with Tukey’s post-test, and those against two groups were made with the Mann–Whitney U test. To determine normality, the Shapiro–Wilk normality test was used. Graphical and statistical analyses were performed with GraphPad Prism 5.0 software (San Diego, CA, USA). Each experiment was performed in duplicate with different culture preparations in at least three independent occasions. Data were represented as mean ± SD. A p < 0.05 is represented as *, p < 0.01 as **, p < 0.001 as ***, and p < 0.0001 as ****; p < 0.05 was the minimum level regarded as a statistically significant difference between groups.

## Results

### Surface expression of HIV receptors and co-receptors on MSCs and adipocytes

**Figure 1:**
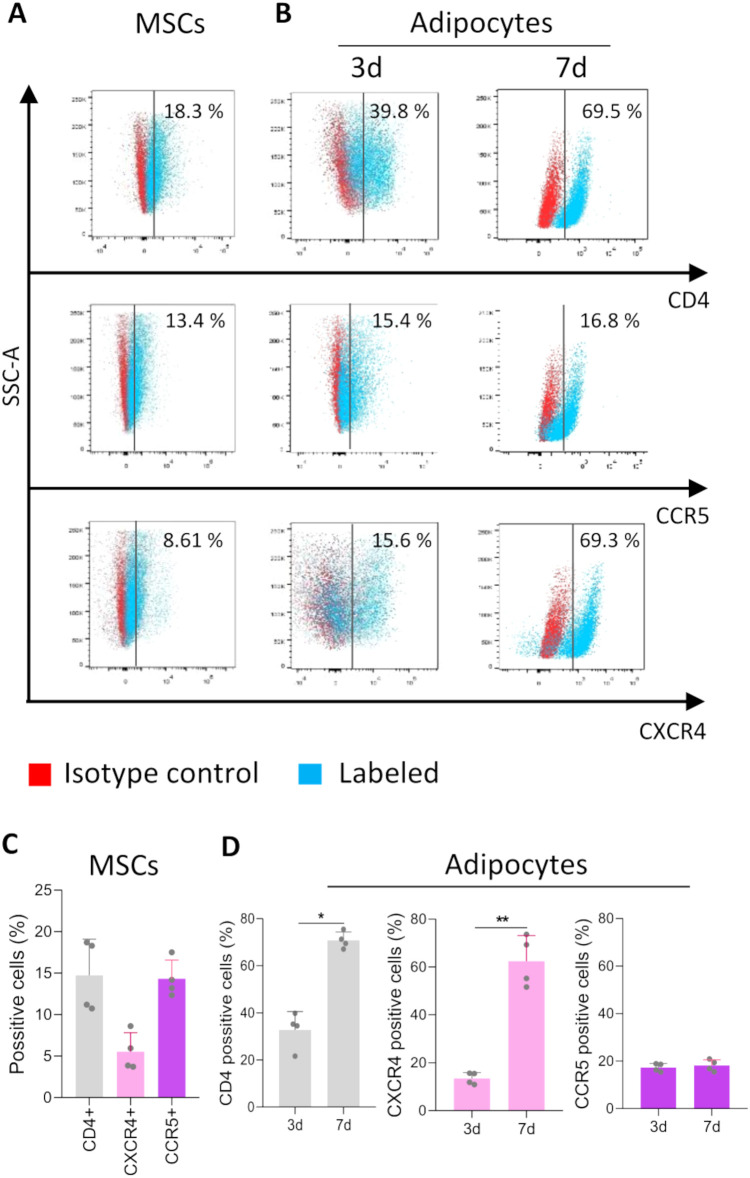
The expression of CD4, CCR5, and CXCR4 on the cell surface and its relation to HIV exposure. Expression of CD4, CCR5, and CXCR4 on the cell surface. Representative dot plots obtained by flow cytometry show the surface expression of CD4, CCR5, and CXCR4 in MSCs **(A)** and at days 3 and 7 of the adipocyte differentiation process **(B)**. The bars represent the percentage of positive cells in MSCs **(C)** and during adipocyte differentiation. Each dot represents the average of technical replicates from an independent biological experiment (n = 4) **(D)**. Data are presented as mean ± SD. *p < 0.05, **p < 0.01.

The attachment and entry of HIV into susceptible cells depend on the presence of chemokine receptors CCR5 and CXCR4, along with CD4. In our study, we assessed the expression of these receptors on the surface of MSCs derived from the umbilical cord. Our findings revealed that 18.3 ± 3.18% of MSCs expressed CD4, 8.61 ± 0.22% expressed CXCR4, and 13.4 ± 0.63% expressed CCR5 receptors (**Figures 1A, C**).

MSCs exhibit versatility and pluripotency, enabling differentiation into various cell types, including adipocytes. Subsequent experiments aimed to evaluate the potential modulation of HIV receptor expression during the differentiation of adipocytes. During the differentiation process, CXCR4, CCR5, and CD4 expression levels were measured on days 3 and 7. Our findings revealed a significant increase in CXCR4 expression throughout the differentiation, peaking at 7 days (69.3 ± 2,24% of positive cells). In contrast, CCR5 expression remained relatively steady until 7 days of the differentiation process (16.8 ± 2.47% of positive cells). When analyzing CD4 expression, our results indicated a gradual significant increase during differentiation, reaching its maximum levels at 7 days post-differentiation (69.5 ± 1,76%) (**Figures 1B, D**).

Together, these results indicate that the HIV-receptor/coreceptors are expressed differentially among the study cell types, and their expression is temporally dissimilar during adipocyte differentiation and maturation.

### MSCs and adipocytes are susceptible to HIV infection

**Figure 2:**
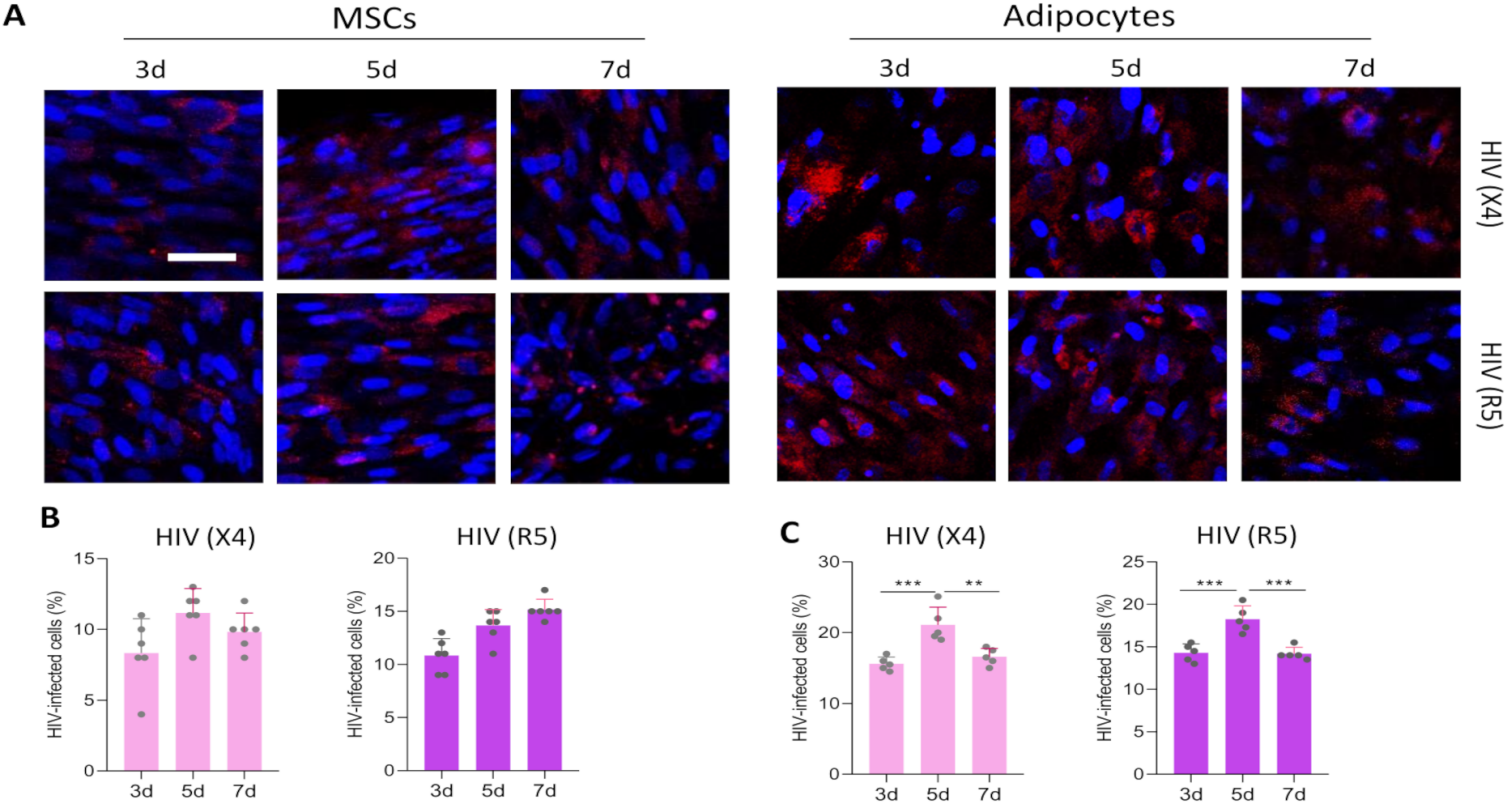
Exposure of MSCs and adipocytes to X4- and R5-tropic HIV. MSCs and 7-day differentiated adipocytes were infected with an inoculum of 1 pg of p24 per cell using CXCR4-tropic HIV (HIV (X4)) and CCR5-tropic HIV (HIV (R5)). Representative microscopy images show the kinetics of HIV replication at 3-, 5-, and 7-days post-infection (d), visualized by immunostaining of the HIV-p24 capsid antigen **(A)**. Bars indicate the percentage of HIV-infected MSCs. Each dot represents the average of technical replicates from an independent biological experiment (n = 6) **(B)** and adipocytes **(C)**. Scale bar: 50 µm. Data are presented as mean ± SD. **p < 0.01, ***p < 0.001.

Experiments were conducted to determine whether MSCs and adipocytes were susceptible and permissive to HIV infection. To assess the susceptibility, the cells were exposed to R5-tropic HIV (AD8) or X4-tropic HIV (NL43) at an inoculum of 1 pg of p24 per cell for 24 hours. Unbound viral particles were extensively washed with the culture medium. At 3-, 5-, and 7-days post-infection, the presence of infected cells was evaluated by confocal microscopy using immunodetection with an antibody targeting the p24 antigen. Our results indicated that MSCs and adipocytes were susceptible to HIV infection (**Figure 2**). However non-significant increase in the percentage of infected cells was observed between 3-, 5- and 7-days post-infection in MSC (**Figure 2B**). In adipocytes, a low increase in the percentage of infected cells was observed at 5 days, albeit with statistical significance, followed by a decrease on day 7 (**Figure 2C**). These results suggest that MSCs and adipocytes were unable to support productive replication of R5- and X4- tropic HIV. However, HIV proviral genome was detected by Alu-qPCR in MSCs and adipocytes (Not shown). These results suggest that although MSCs and adipocytes express HIV receptors and co-receptors and allow viral genome integration, they are unable to support productive replication of both R5- and X4-tropic HIV. This indicates that additional cellular restrictions may prevent efficient viral replication in these cell types.

### HIV modulates adipocyte differentiation

To assess whether infection affects adipocyte differentiation, MSCs were exposed to X4- and R5-tropic HIV in the presence of a differentiation medium. At 7- and 10-days post-differentiation, the presence of adipocytes was revealed by staining lipid droplets with Bodipy 493/503.

**Figure 3:**
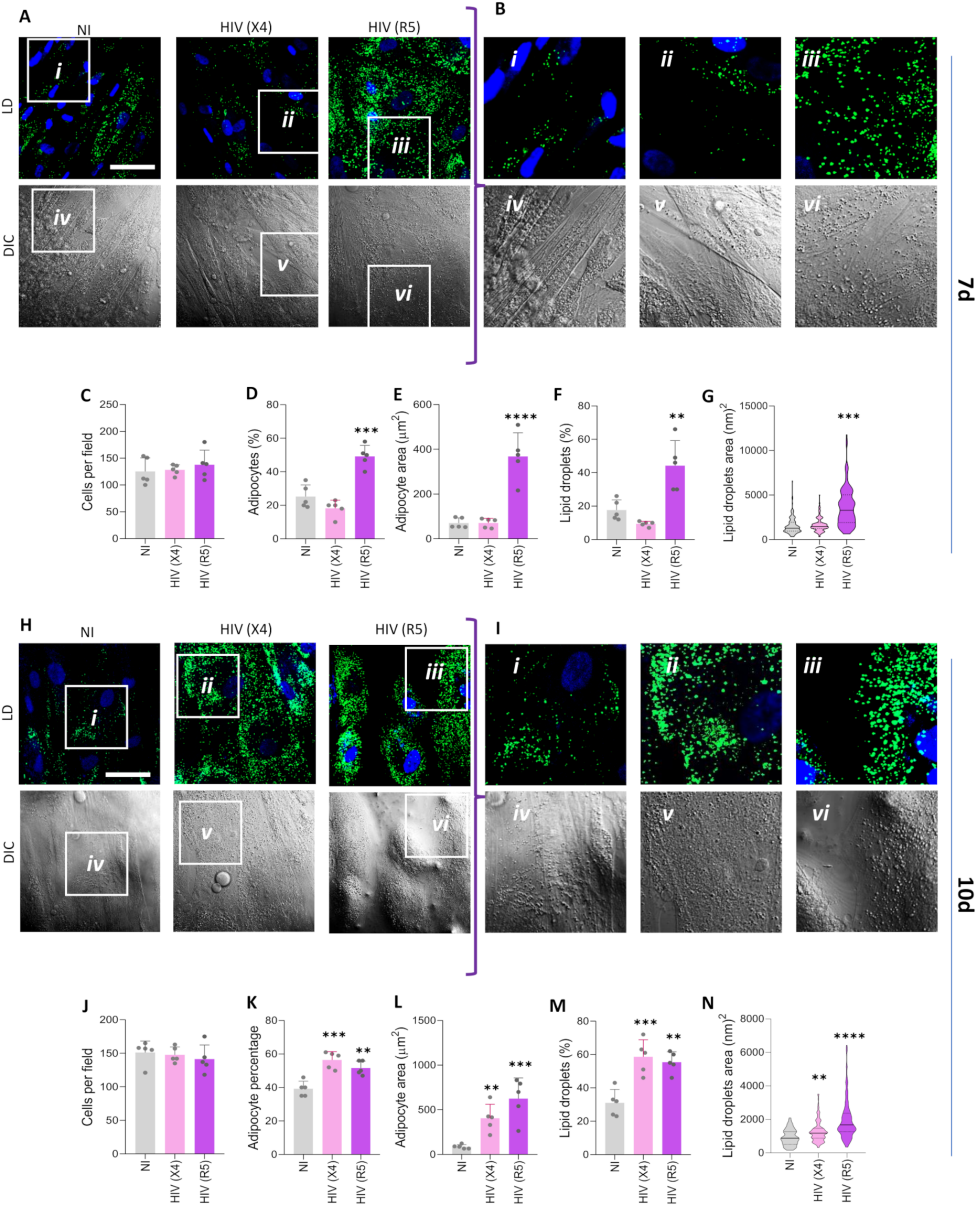
HIV modulates adipocyte differentiation. Effect of CXCR4-tropic HIV [HIV (X4)] and CCR5-tropic HIV [HIV (R5)] exposure on adipocyte differentiation. Representative microscopy images show the presence of lipid droplets stained with Bodipy 493/503 (LD) and Differential Interference Contrast (DIC) at 7 **(A)** and 10 **(H)** days post-differentiation. **(B, I)** Magnified insets from panels A and H, respectively. Quantification of the experiments shown in panels A and H includes the number of cells per field **(C, J)**, percentage of adipocytes per field **(D, K)**, adipocyte area **(E, L)**, percentage of lipid droplets per field **(F, M)** Scale bar: 50 µm. Data are presented as mean ± SD. Each dot represents the average of technical replicates from an independent biological experiment (n = 5). **p < 0.01, ***p < 0.001 and ****p < 0.0001 compared to non-infected cells (NI).

At 7 days post-differentiation, our results indicated that R5-tropic HIV increased adipocyte differentiation, evidenced by a significant rise in the number and size of differentiated adipocytes and an increase in both the number and size of lipid droplets. This occurred without affecting the total cell count compared with uninfected cells. In contrast, X4-tropic HIV did not modulate adipocyte differentiation compared to uninfected controls (**Figures 3A–G**).

At 10 days post-differentiation, both viral tropisms induced an increase in adipocyte differentiation, accompanied by an increase in the number and size of lipid droplets, without affecting the total cell count compared to uninfected cells (**Figures 3H–N**).

Given the ability of HIV to stimulate adipocyte differentiation, we conducted subsequent experiments to determine whether infection could also modulate the transcription of essential adipogenic factors, namely *PPAR-γ*, *C/EBPα*, and *C/EBPβ* (Darlington et al., 1998). MSCs were infected, incubated with adipocyte differentiation medium, and mRNA levels of the mentioned factors were measured at 1-, 3-, 7- and 10-days post-differentiation.

**Figure 4:**
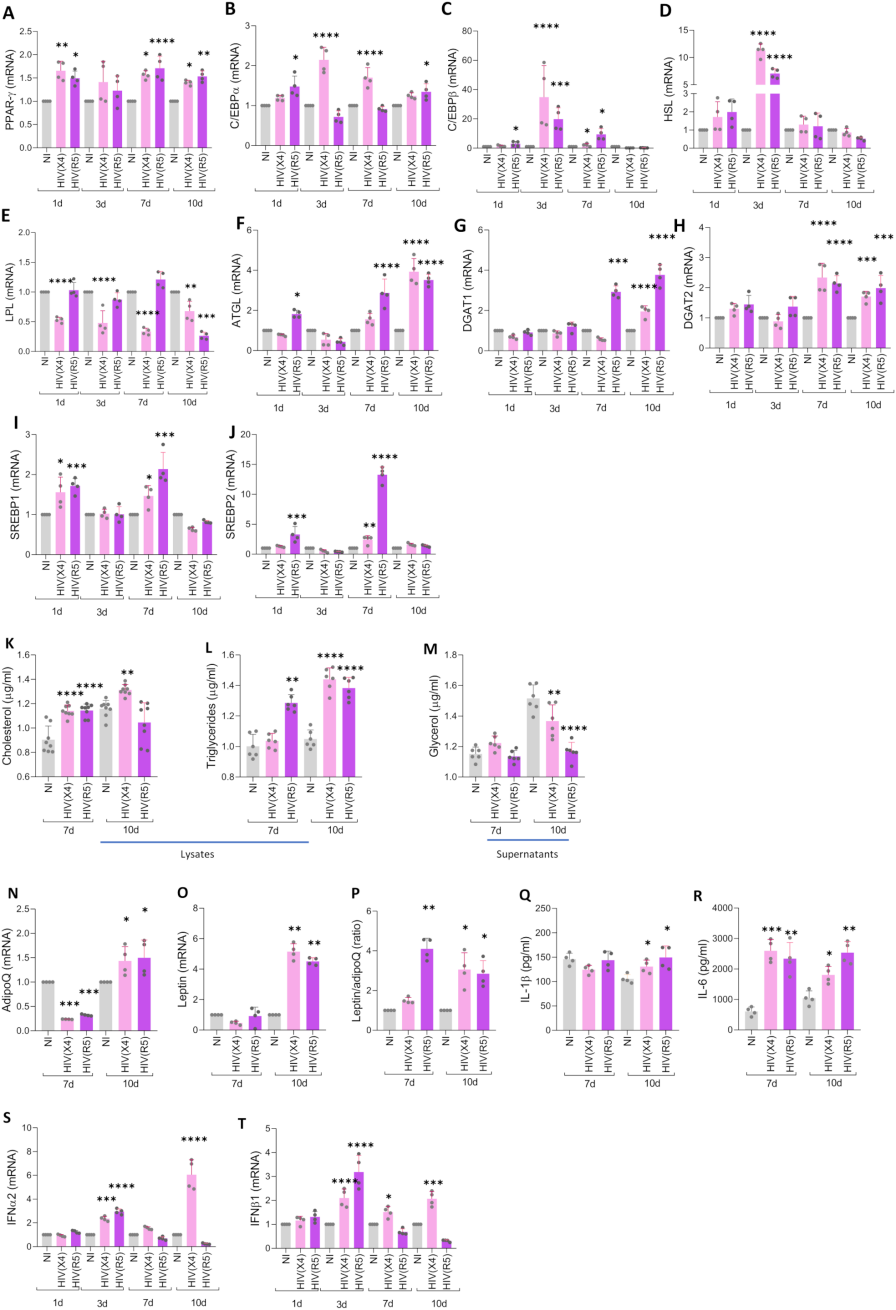
HIV modulates adipocyte mediators during its differentiation. Effect of HIV infection on the expression of *PPAR-γ* **(A)**, *C/EBPα* **(B)**, *C/EBPβ* **(C)**, *HSL* **(D)**, *LPL* **(E)**, *ATGL* **(F)**, *DGAT1* **(G)**, *DGAT2* **(H)**, *SREBP1* **(I)**, and *SREBP2* **(J)**, as determined by RT-qPCR at days 1, 3, 7, and 10 post-differentiation. Cholesterol **(K)** and triglycerides **(L)** were measured in cell lysates, while glycerol release was measured in culture supernatants **(M)**. *ADIPOQ* **(N)** and *LEPTIN* **(O)** transcription levels were assessed by RT-qPCR, and the *LEPTIN/ADIPOQ* ratio was calculated **(P)**. IL-1β **(Q)** and IL-6 **(R)** secretion levels were measured in culture supernatants using ELISA. The analyses in panels K–R were performed at days 7 and 10 post-infection. *IFN-α2* **(S)** and *IFN-β1* **(T)** transcription was determined by RT-qPCR at days 1, 3, 7, and 10 post-differentiation. Data are presented as mean ± SD. Each dot represents the average of technical replicates from an independent biological experiment (n = 4). *p < 0.05, **p < 0.01, ***p < 0.001, ****p < 0.0001 compared to non-infected cells (NI).

Our results (**Figure 4**; **Supplementary Figure 1**) indicated that both HIV tropisms can induce *PPAR-γ* upregulation at 1- 7- and 10-days post-differentiation compared to uninfected controls (**Figure 4A**). The analysis of *C/EBPα* levels revealed that R5-tropic HIV induces its increase only at 1- and 10-days post-infection, whereas X4-tropic virus induces the increase at 3- and 7-days post-differentiation respect to uninfected controls (**Figure 4B**). In contrast, the analysis of *C/EBPβ* showed that R5-tropic HIV induces a rise in mRNA levels at 1-, 3-, and 7-days post-differentiation, while X4-tropic HIV induces an increase in *C/EBPβ* only at 3- and 7-days post-differentiation in comparison with uninfected cells (**Figure 4C**). These findings suggest that HIV infection can modulate adipocyte differentiation in a tropism-dependent manner. While R5-tropic HIV promotes early and sustained adipocyte differentiation, X4-tropic HIV appears to exert a delayed effect. The upregulation of key adipogenic transcription factors, particularly *PPAR-γ*, *C/EBPα*, and *C/EBPβ*, may underlie these differences. The distinct temporal patterns of *C/EBPα* and *C/EBPβ* expression induced by each viral tropism could contribute to the differential regulation of adipogenesis, highlighting a potential role for HIV in altering adipose tissue homeostasis.

### Lipogenesis and Lipolysis upregulation are related to increased lipid droplets size during HIV-induced adipocyte differentiation

To investigate whether the increase in lipid droplet number and size was linked to altered lipogenesis and adipogenesis, we examined the mRNA expression of key lipogenic and lipolytic enzymes—including DGAT1 and DGAT2, which are critical for triglyceride synthesis and lipid droplet expansion—during adipocyte differentiation in the presence of HIV. We assessed the mRNA transcription of lipolytic and lipogenic enzymes during adipocyte differentiation in the presence of HIV. Our results revealed that *HSL* was significantly upregulated three days after infection with both X4 and R5 tropic HIV (**Figure 4D**). In contrast, *LPL* expression was significantly downregulated in MSCs differentiated in the presence of X4-tropic HIV at 1, 3, 7, and 10 days post-differentiation. While, R5-tropic HIV induced *LPL* downregulation only at 10 days post-differentiation, compared to uninfected cells (**Figure 4E**). Additionally, *ATGL* was upregulated at days 1, 7, and 10 post-differentiation in MSCs exposed to R5-tropic HIV, while X4-tropic HIV only upregulated *ATGL* expression at day 10 (**Figure 4F**).

DGAT1 and DGAT2, key enzymes involved in triglyceride synthesis, were not modulated by X4- or R5-tropic HIV at 1- and 3- days post-differentiation, according to our results. However, at 7- and 10-days post-differentiation, R5-tropic HIV induced a significant increase in *DGAT1* whereas X4-tropic HIV upregulate *DGAT1* only at 10 days post differentiation. Both viral tropisms significantly upregulated *DGAT2* at days 7 and 10, coinciding with the increase in the size of lipid droplets compared to uninfected controls (**Figures 4G, H**). *SREBP-1*, a transcription factor critical for fatty acid and triglyceride synthesis, was significantly upregulated at days 1 and 7 in cells exposed to both viral strains (**Figure 4I**), indicating early activation of the lipogenic program.

*SREBP-2*, primarily associated with cholesterol metabolism, was also upregulated by R5-HIV at days 1 and 7, whereas X4-HIV induced a moderate increase only at day 7 (**Figure 4J**). The timing of *SREBP-2* upregulation also coincided with lipid droplet hypertrophy.

Collectively, these results indicate that HIV infection modulates lipid homeostasis by upregulating lipolytic and lipogenic pathways in a tropism- and time-dependent manner.

As a result, at 7 days post-differentiation, HIV (R5- and X4- tropic) induces an increase in intracellular cholesterol compared to uninfected controls, and it remains elevated only in comparison to uninfected controls when exposed to HIV-X4 (**Figure 4K**). Additionally, an increase in intracellular triglycerides was observed in cell lysates from cells infected with HIV (X4 and R5), along with a concomitant reduction in glycerol presence in culture supernatants (**Figures 4L, M**).

Adipocyte hypertrophy is linked to an enhanced inflammatory profile (Gao et al., 2025).In line with this our findings show that both HIV tropisms increase the *LEPTIN/ADIPONECTIN* ratio (**Figures 4N–P**) and stimulate IL-1β secretion at 10 days post-infection (**Figure 4Q**). Additionally, both viral strains induce elevated IL-6 secretion at 7 and 10 days post-infection (**Figure 4R**); although IL-6 is commonly associated with inflammation, it also plays a role in metabolic regulation and can promote adipocyte browning under specific conditions (Radvanyi and Roszer, 2024).

Notably, TNF-α production was not stimulated by HIV infection (not shown). Collectively, our results suggest that HIV induces adipocyte hypertrophy in a tropism-dependent manner, characterized by increased lipid accumulation and a proinflammatory profile.

### X4- or R5- tropic HIV infected adipocytes induce IFN-α2 and IFN-β1

Previous studies have shown that downregulation of *SREBP2*, a key regulator of cholesterol biosynthesis, enhances the expression of type I interferons and interferon-stimulated genes (ISGs) (York et al., 2015). Conversely, activation of the cholesterol biosynthetic pathway may dampen the type I IFN response, thereby hindering the elimination of infectious viruses. Given the observed modulation of *SREBP2* during HIV-induced adipocyte differentiation, we next investigated whether HIV infection alters the expression of type I *IFN*s. We quantified the mRNA levels of *IFN-α2* and *IFN-β1* at various time points during adipocyte differentiation in the presence or absence of X4- or R5-tropic HIV. Our findings revealed that *IFNα2* and *IFNβ1* mRNA transcription fluctuated following HIV exposure. Specifically, their expression increased at 3 days post-differentiation in the presence of both X4- and R5-tropic HIV compared to uninfected controls. By 7- and 10-days post-differentiation*, IFNα2* and *IFNβ1* levels decreased but remained significantly elevated in cells differentiated with X4-tropic HIV compared to uninfected controls (**Figures 4S, T**). In contrast, *IFN* expression in R5-HIV-infected cells returned to baseline by day 10.

These findings suggest that HIV infection triggers a transient activation of type I IFN responses during early adipocyte differentiation, with a more sustained effect observed in X4-HIV-infected cells.

### Lipid droplets-lysosome interaction during adipocyte differentiation under X4- and R5- tropic HIV infection

**Figure 5:**
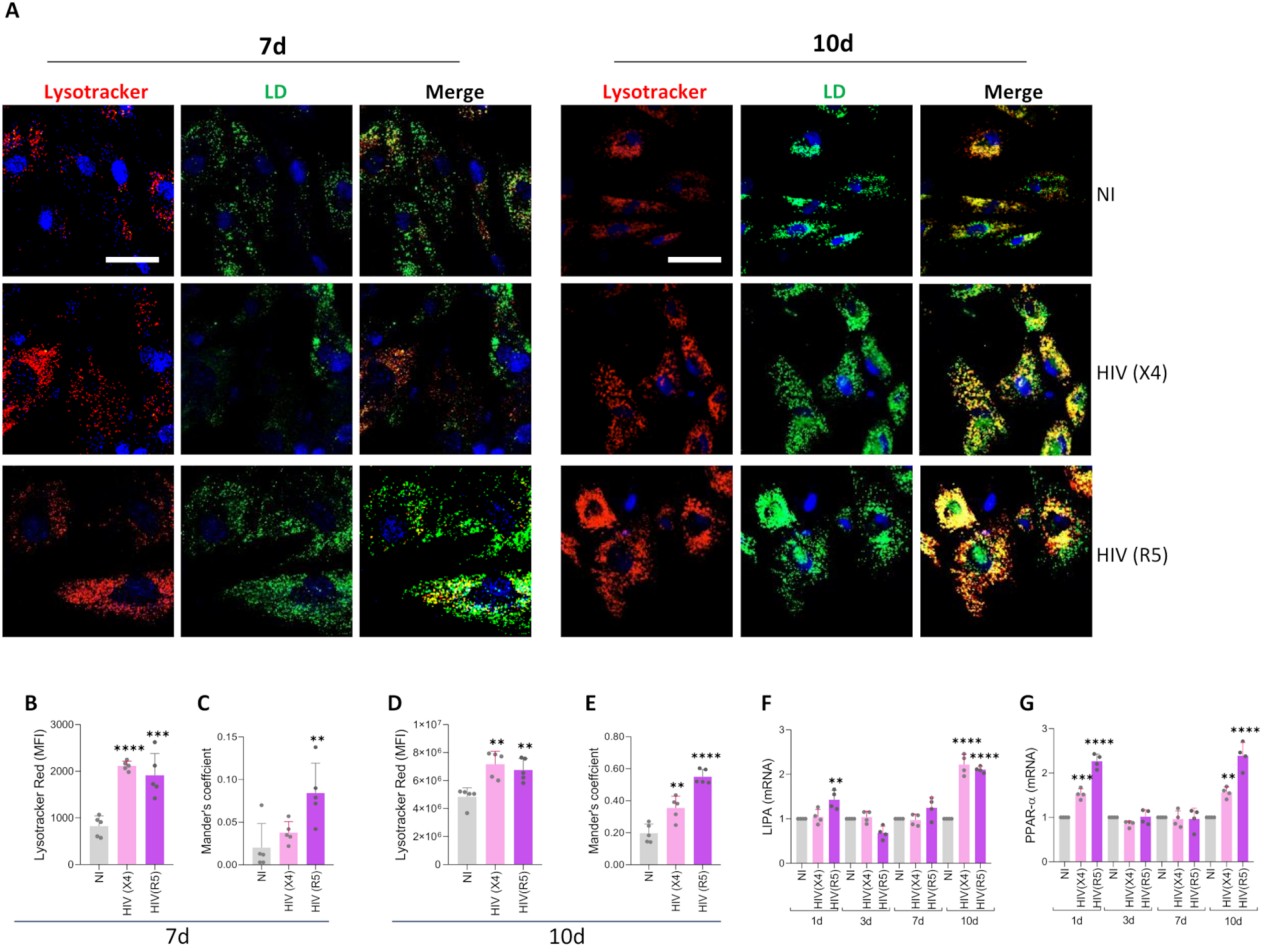
Lipid droplets-lysosome interaction during adipocyte differentiation under X4- and R5- tropic HIV infection. Representative images showing lysosomes stained with LysoTracker Red and lipid droplets (LD) stained with Bodipy 493/503 at 7- and 10-days post-differentiation in cells exposed to HIV (X4 and R5 strains) **(A)**. Quantification of median fluorescence intensity (MFI) for lysosomal content was performed using ImageJ from the images in panel A at 7 **(B)** and 10 **(D)** days post-differentiation. Lipid droplet–lysosome colocalization was analyzed using Mander’s overlap coefficient at 7 **(C)** and 10 **(E)** days post-differentiation. *LIPA* **(F)** and *PPAR-α* **(G)** transcription were measured by RT-qPCR at 1-, 3-, 7- and 10-days post-differentiation. Scale bar: 50 µm. Data are presented as mean ± SD. Each dot represents the average of technical replicates from an independent biological experiment (n = 5). **p < 0.01; ***p < 0.001; ****p < 0.0001 compared to non-infected cells (NI).

Organelle interactions have emerged as critical regulators of intracellular lipid trafficking and metabolism. To evaluate lysosomal dynamics during HIV infection, we used Lysotracker, a fluorescent probe, to monitor lysosomal activity via confocal microscopy (**Figure 5**). Our analysis revealed that both X4- and R5-tropic HIV infections increased lysosomal activity in differentiating adipocytes, notably at 7- and 10-days post-infection (**Figures 5B, D**). To investigate whether HIV infection promotes lipid droplet–lysosome interaction, we performed colocalization analysis using fluorescent labeling of lipid droplets and lysosomes. Confocal imaging showed evident colocalization in infected adipocytes, with temporal differences depending on HIV tropism. R5-tropic HIV induced peak colocalization at 7 days, whereas X4-tropic HIV showed maximal interaction at 10 days post-infection (**Figures 5C, E**). This suggests that acid lipolysis is dynamically regulated during HIV infection and may contribute to lipid remodeling in a tropism-specific manner.

Lysosomal acid lipase (LAL), encoded by the *LIPA* gene, is the sole enzyme identified to function at acidic pH within the lysosome. Consistently, we observed significant upregulation of *LIPA* mRNA expression in adipocytes infected with either HIV strain at 10 days post-differentiation (**Figure 5F**), further supporting the activation of lysosomal lipid degradation pathways.

LIPA is regulated by *PPAR-α* which was significantly upregulated at both day 1 and day 10 post-differentiation in adipocytes infected with either X4- or R5-tropic HIV (**Figure 5G**), indicating a coordinated transcriptional program promoting lysosomal lipolysis and mitochondrial fatty acid oxidation.

Together, these findings suggest that HIV infection enhances lysosomal function and promotes lipid droplet–lysosome interaction in differentiating adipocytes, potentially facilitating lipid mobilization through acid lipolysis.

### Interaction between lipid droplets and mitochondria during adipocyte differentiation under X4- and R5-tropic HIV infection

Mitochondria are central regulators of cellular metabolism and play a key role in lipid catabolism. In turn, mitochondrial function supports cellular energy demands and may contribute indirectly to lipid droplet growth by fueling lipogenic processes.

To investigate the relationship between mitochondria and lipid droplets during HIV-induced adipocyte differentiation, we used MitoTracker to assess mitochondrial mass and colocalization with lipid droplets at days 7 and 10 post-differentiation time points that coincided with the observed increase in lipid droplet size.

**Figure 6:**
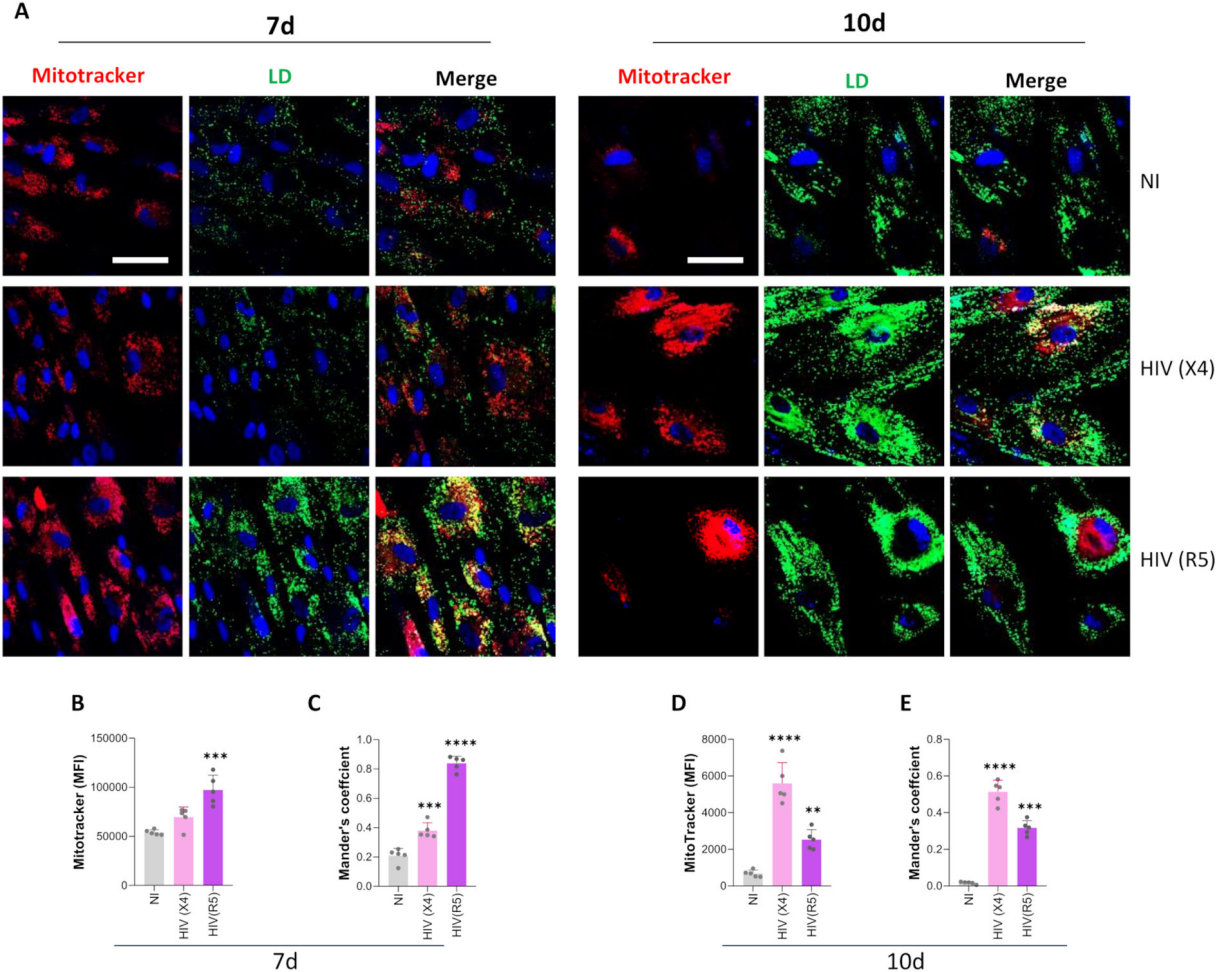
Interaction between lipid droplets and mitochondria during adipocyte differentiation under X4- and R5-tropic HIV infection. Representative images showing mitochondria stained with Mitotracker Deep Red and lipid droplets (LD) stained with Bodipy 493/503 at 7- and 10-days post-differentiation in cells exposed to HIV (X4 and R5 strains) **(A)**. Quantification of median fluorescence intensity (MFI) for mitochondrial content was performed using ImageJ from the images in panel A at 7 **(B)** and 10 **(D)** days post-differentiation. Lipid droplet–mitochondria colocalization was analyzed using Mander’s overlap coefficient at 7 **(C)** and 10 **(E)** days post-differentiation. Scale bar: 50 µm. Data are presented as mean ± SD. Each dot represents the average of technical replicates from an independent biological experiment (n = 5). **p < 0.005; ***p < 0.001; ****p < 0.0001 compared to non-infected cells (NI).

At day 7, R5-tropic HIV significantly increased mitochondrial mass, whereas X4-tropic HIV did not (**Figures 6A, B**). However, both tropisms promoted enhanced colocalization between mitochondria and lipid droplets, suggesting early activation of lipid trafficking between these organelles (**Figures 6A, C**).

By day 10, both viral tropisms induced a significant increase in mitochondrial mass and also in mitochondria and lipid droplets colocalization compared to uninfected controls (**Figures 6A, D, E**). These findings indicate that HIV infection promotes mitochondrial biogenesis and enhances physical interactions between mitochondria and lipid droplets during adipocyte differentiation.

### HIV was not able to modulate mROS production during adipocyte differentiation

To determine whether the observed increase in mitochondrial mass corresponded to enhanced oxidative metabolism or stress, we measured mROS production during adipocyte differentiation in the presence or absence of X4- or R5-tropic HIV.

**Figure 7:**
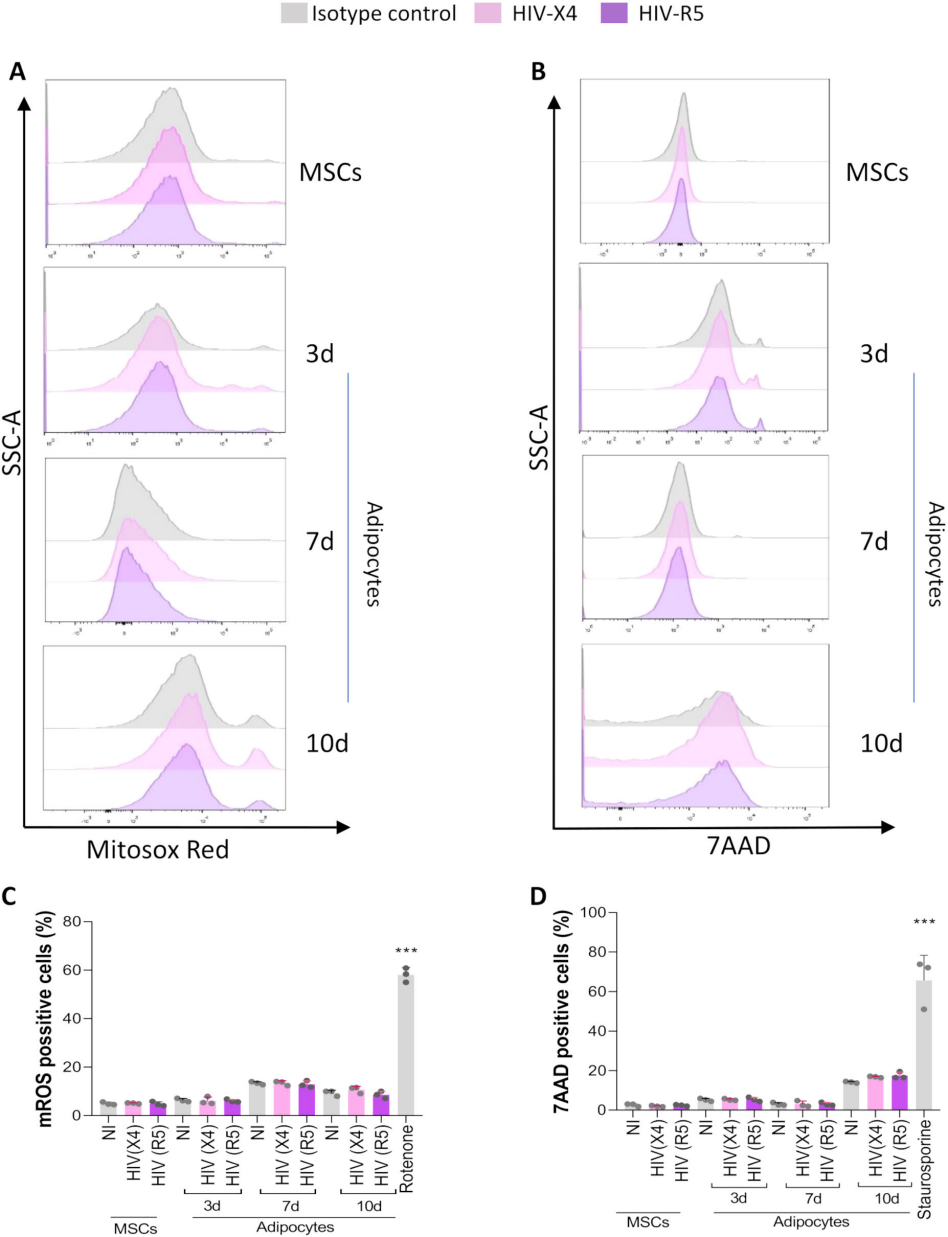
Effects of HIV on cell viability and mROS production. Representative flow cytometry histograms of MitoSOX staining, illustrating mROS un MSCs and at 1,3 and 7 days (d) post-adipocyte differentiation, in cells exposed to HIV (X4 and R5 strains) **(A)**. Representative flow cytometry histograms of 7AAD staining, illustrating cell death un MSCs and at 3, 7 and 10 days (d) post-adipocyte differentiation **(B)**. Quantification of mROS production presented in panel A, expressed as the percentage of cells stained with MitoSOX Red **(C)**. Assessment of cell viability, shown as the percentage of cells stained with 7AAD at the conditions at time points presented in panel B **(D)**. NI: non-infected; Rotenone and staurosporine were used as positive control of mROS production and cell death, respectively. Data are presented as mean ± SD. Each dot represents the average of technical replicates from an independent biological experiment (n = 3). ***p < 0.001compared to non-infected cells (NI).

Our results showed that neither X4- nor R5-tropic HIV infection significantly increased mROS levels compared to uninfected controls at 3-, 7-, or 10-days post-differentiation (**Figures 7A, C**). Additionally, no significant changes in cell viability were observed under any condition (**Figures 7B, D**).

These findings indicate that while HIV infection promotes mitochondrial biogenesis and organelle interaction with lipid droplets, it does not induce oxidative stress or compromise cell viability during adipocyte differentiation. This suggests that mitochondrial remodeling under these conditions may support metabolic adaptation rather than reflecting mitochondrial dysfunction.

### Role of CXCR4 and CCR5 in the increase of lipid droplets size induced by X4- and R5-tropic HIV infection

CXCR4 and CCR5 have been associated with adipogenesis and adipose tissue remodeling, suggesting roles in differentiation and inflammation during adipocyte development. To assess the specific contributions of CXCR4 and CCR5 in HIV-mediated modulation of adipocyte differentiation, we treated MSCs with X4- or R5-tropic HIV during differentiation in the presence or absence of the CXCR4 antagonist AMD3100 or the CCR5 antagonist TAK799.

**Figure 8:**
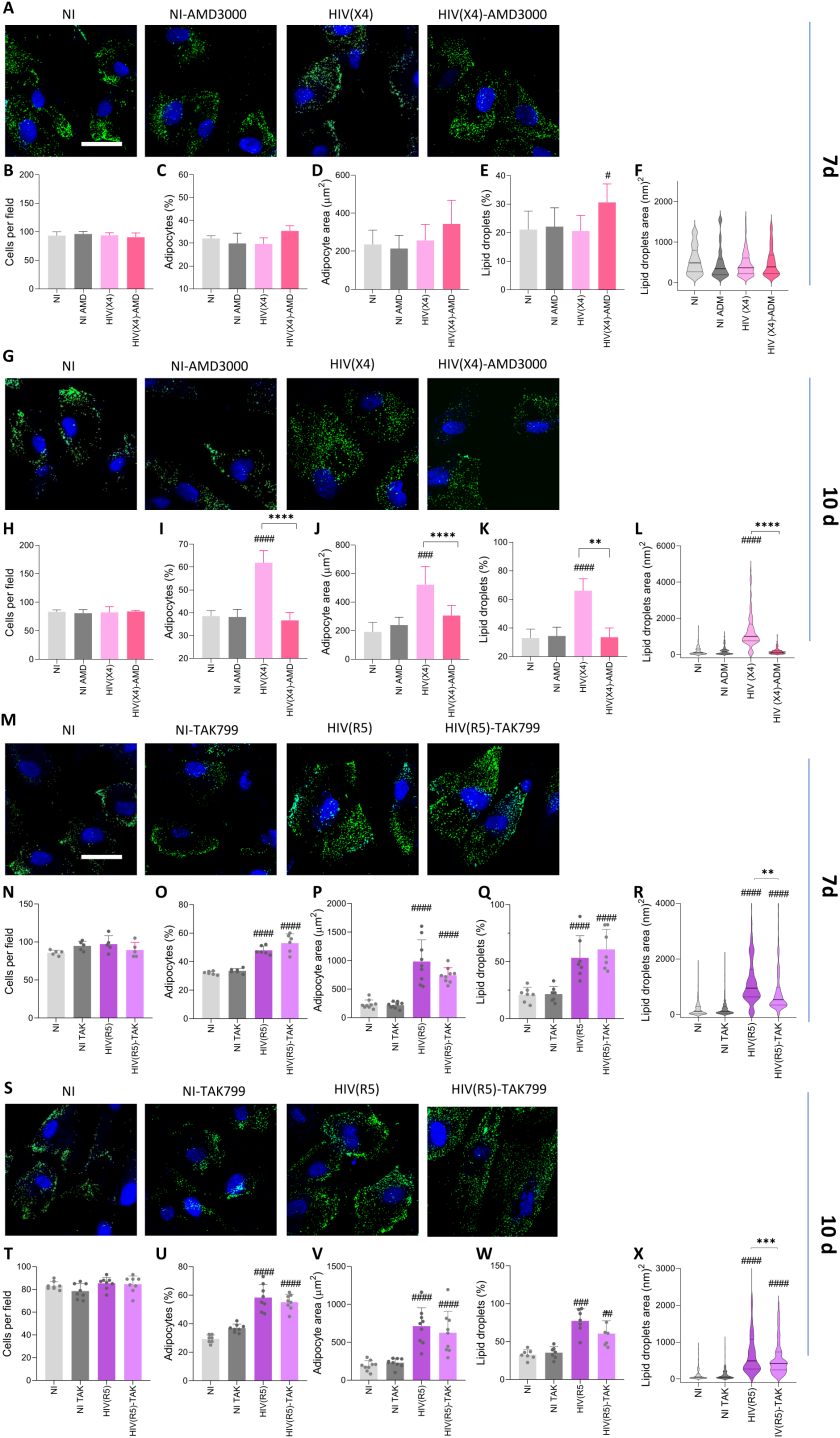
Role of CCR5 and CXCR4 in adipocyte differentiation. Effect of CXCR4-tropic HIV (HIV (X4)), HIV-X4 + AMD3100, CCR5-tropic HIV (HIV (R5)), and HIV-R5 + TAK-779 exposure on adipocyte differentiation. Representative microscopy images show the presence of lipid droplets stained with Bodipy 493/503 (LD) at 7 **(A, M)** and 10 **(G, S)** days post-differentiation. Quantification of the experiments shown in panels **(A, F, M, S)** includes the number of cells per field **(B, H, N, T)**, percentage of adipocytes per field **(C, I, O, U)**, adipocyte area **(D, J, P, V)**, percentage of lipid droplets per field **(E, K, Q, W)** and lipid droplets size **(F, L, R, X)**. Scale bar: 50 µm. Data are presented as mean ± SD. Each dot represents the average of technical replicates from an independent biological experiment (n = 6). ^##^p < 0.01, ^###^p < 0.001 and ^####^p < 0.0001 compared to non-infected cells (NI). *p < 0.05 **p < 0.01, and ****p < 0.0001.

In the presence of X4-tropic HIV, CXCR4 inhibition by AMD3100 significantly reduced adipocyte number, cell size, and lipid droplet accumulation to levels comparable to uninfected controls at 10 days post-differentiation. No significant effects were observed at 7 days post-differentiation (**Figures 8A–L**). Conversely, CCR5 inhibition with TAK779 did not affect adipocyte number or lipid droplet accumulation in cells exposed to R5-tropic HIV. However, TAK779 treatment significantly altered lipid droplet size in these cells at 7 and 10 days post-differentiation (**Figures 8M-X**). Importantly, treatment with AMD3100 or TAK799 alone did not affect adipocyte differentiation, highlighting the specificity of the CXCR4-dependent effect in the context of X4-HIV exposure.

These findings identify CXCR4 as a critical mediator of HIV-induced adipocyte hypertrophy and lipid droplet enlargement during X4-tropic HIV infection, whereas CCR5 does not appear to play a direct role in modulating these processes during R5-tropic HIV exposure.

## Discussion

People living with HIV (PLWH) frequently exhibit reduced bone mineral density (BMD), predisposing individuals to conditions like osteopenia and osteoporosis. This heightened susceptibility to bone fragility increases the likelihood of fractures compared to those in the general population (Cotter and Mallon, 2014).

In bone diseases, a pathological decline in BMD often occurs alongside increased fat deposition within the marrow and adjacent tissues (Meunier et al., 1971; Verma et al., 2002; Yeung et al., 2005).

In our laboratory, we have shown that HIV exposure during MSC‐to‐osteoblast differentiation does not impair matrix deposition; however, it markedly elevates RANKL production and vitronectin accumulation, implying a potential shift toward enhanced osteoclastogenesis and altered bone homeostasis (Freiberger et al., 2024). Building on this inverse relationship between osteogenesis and marrow adiposity, we therefore set out to determine whether HIV also influences MSC differentiation into adipocytes.

Notably, although adipocytes derived from MSCs permitted viral entry, they were non-permissive to productive HIV infection. This result is consistent with previous studies showing that, while HIV can enter adipocytes, these cells are unable to support productive viral replication (Munier et al., 2003; Maurin et al., 2005). In those studies, a peak in viral protein production was observed at three days post−infection, followed by a gradual decline. This phenomenon was not prevented by HIV inhibitors, suggesting passive release of viral antigens rather than active replication (Maurin et al., 2005). Furthermore, Munier et al, demonstrated using VSV−G pseudotyped viral particles that viral entry is the limiting step in these cells (Munier et al., 2003). In contrast, we found that adipocytes differentiated from MSCs were not productively infected, even by X4–tropic HIV, despite expressing high surface levels of CD4 and CXCR4. This indicates a non–permissive replication environment, possibly due to the activation of intrinsic restriction factors upon CD4 and co–receptor engagement during viral entry.

Nonetheless, both R5- and X4-tropic HIV strains induced a significant increase in adipocyte number, lipid droplet count, and size. Notably, R5-tropic HIV induced these changes earlier than the X4-tropic strain.

Differences in adipocyte differentiation dynamics may be linked to receptor expression patterns. While CCR5 remained stable, CXCR4 was markedly upregulated by day 7, potentially driving the increased adipocyte number and lipid accumulation by day 10.

Multiple transcription factors, including *PPAR-γ*, *C/EBPα*, and *C/EBPβ*, are key regulators of adipogenesis. *PPAR-γ* plays a central role, and its upregulation in response to both viral tropisms aligns with findings from *in vitro* models of infectious diseases (Ayyappan et al., 2019; Gonzalez et al., 2019; Pesce Viglietti et al., 2020). *C/EBPα* is expressed in the late phase of adipocyte differentiation, while *C/EBPβ* is expressed very early during adipogenesis. Additionally, it is necessary for sustaining the expression of *PPAR-γ* and *C/EBPα* (Darlington et al., 1998). Both transcription factors were upregulated in response to HIV exposure, though with temporal and tropism-specific differences. These variations may be attributed to post-transcriptional modifications that regulate their activity (Hattori et al., 2003; Cesena et al., 2007; Nerlov, 2008), and potentially to compensatory mechanisms between *C/EBPα* and *C/EBPβ* (Lefterova et al., 2008).

Adipocyte enlargement is associated with increased lipid droplet size, necessitating enhanced protein and membrane synthesis, as well as coordinated lipogenesis and lipolysis (Laurencikiene et al., 2011).

*ATGL* and *HSL* are the major rate-determining enzymes for lipolysis in adipocytes. They are involved in the hydrolysis of triglycerides, releasing fatty acids essential as energy substrates, precursors for membrane lipid synthesis, and ligands for nuclear receptors (Miyoshi et al., 2008; Schreiber et al., 2019). In our study, *ATGL* expression increased in adipocytes derived from R5-HIV–infected MSCs, corresponding with larger lipid droplets. Conversely, *HSL* expression decreased at day 10 post-differentiation in cells exposed to both viral tropisms. *LPL*, which facilitates the uptake of fatty acids from circulating triglycerides (Weinstock et al., 1997; Zechner et al., 2000), was downregulated at early stages of differentiation in X4–HIV–infected cells. This reduction may have limited lipid accumulation at early time points in these cells.

In more recent findings, a notable function of acid lipolysis has emerged, implicating the involvement of lysosomal lipases in the breakdown of lipid droplets, operating alongside cytoplasmic lipases (Yu and Li, 2017). Here, we have found that X4 and R5 tropic HIV induces an increase in lipid droplet-lysosome interaction, suggesting that acid lipolysis could play a role in the degradation of lipid droplets. The *LIPA* gene, which encodes LAL, was upregulated in cells exposed to X4- and R5-tropic HIV (Li and Zhang, 2019).

LIPA (LAL) is *PPARα*−regulated (Bougarne et al., 2018), and HIV (X4/R5) induced its upregulation alongside elevated PPARα at days 1 and 10 post−differentiation, suggesting that PPARγ activation, more than mere overexpression, may underlie this increase.

Triglyceride synthesis is catalyzed by the DGAT family of enzymes (Liu et al., 2012; Chitraju et al., 2017). *DGAT1*, localized to the ER, prevents lipid-induced ER stress (Chitraju et al., 2017), and its expression was increased in R5–HIV–infected cells at day 7 and in both tropisms by day 10. On the other hand, *DGAT2* is implicated in increasing lipid droplet size (Zhang et al., 2014), was upregulated at all time points post differentiation in response to both HIV strains. In X4-HIV–infected cells, *DGAT2* upregulation may act as a compensatory mechanism in response to reduced *LPL* expression.

Lipid biosynthesis is also regulated by *SREBPs*, which control fatty acid (*SREBP1a/c*) and cholesterol (*SREBP2*) synthesis (Horton et al., 2002a, 2002b; Iizuka et al., 2004). Moreover, *SREBP-2* transcription factor is a common feature of hypertrophied adipocytes (Bauer et al., 2011). The observed increase in triglyceride and cholesterol production in infected cells suggests the active involvement of both *SREBP* pathways.

As previously reported, type I interferons are linked to cholesterol metabolism (York et al., 2015). Accordingly, we observed that increases in IFNα2 and IFNβ1 transcription in infected adipocytes coincided with periods when SREBP2 was not upregulated.

Lipid droplet–mitochondria contact sites are critical for lipid storage and β-oxidation (Liao et al., 2022). HIV exposure increased lipid droplet–mitochondria contacts and mitochondrial mass in adipocytes. Although mROS function as adipogenic signaling molecules (Orava et al., 2011), their levels remained unchanged in infected cells, implying that mitochondrial expansion occurred without increased oxidative stress.

Our results also highlight the distinct roles of CXCR4 and CCR5 in adipocyte differentiation. CXCR4 expression increased during adipogenesis and was associated with *PPAR-γ* expression (Tontonoz et al., 1994; Steiner et al., 2024). Blocking CXCR4 with AMD3100 during differentiation in the presence of X4-tropic HIV significantly reduced adipocyte number, size, and lipid droplet accumulation, reinforcing its role in this process. Conversely, CCR5, primarily linked to adipose tissue inflammation (Mello Coelho et al., 2009; Kitade et al., 2012), did not appear to influence adipocyte differentiation under R5-tropic HIV exposure, as TAK799 had no observable effect. These results suggest that CXCR4 is essential for adipocyte differentiation, whereas CCR5 may be more involved in adipose tissue homeostasis and inflammation.

In summary, our findings demonstrate that exposure of MSCs to HIV can modulate adipocyte differentiation—a process essential for maintaining bone–fat homeostasis. These alterations may underlie the metabolic disturbances and bone loss commonly observed in people living with HIV. In line with this, early metabolic changes seen in untreated patients suggest that HIV-1 proteins may directly contribute to adipocyte dysfunction, as reported in cases of HIV-associated lipodystrophy (Giralt et al., 2011). Fat redistribution and metabolic abnormalities in PLWH are known to be complex and multifactorial, involving not only direct effects of viral proteins and antiretroviral therapy on adipocyte health, but also genetic factors, microbial translocation, immune dysregulation, chronic inflammation, and accelerated fibrosis (Koethe et al., 2020). Further research is needed to better understand how HIV influences inflammatory responses, metabolic regulation, and the development of dyslipidemia.

This study has limitations. The experiments were conducted *in vitro* using umbilical cord–derived MSCs, which, although a useful model for studying adipocyte differentiation, might not fully recapitulate the complexity of adult adipose tissue or the *in vivo* environment. Furthermore, the study focused on a limited set of viral strains (R5- and X4-tropic HIV) and did not address how other viral variants or the presence of antiretroviral therapy might modify adipocyte differentiation and function. Although the investigation provided insights into changes in key adipogenic and lipolytic factors and highlighted the role of CXCR4, the precise molecular mechanisms underlying these alterations and their broader implications for metabolic dysregulation are not completely defined.

## Data Availability

The raw data supporting the conclusions of this article will be made available by the authors, without undue reservation.
