# Supplementary material for: HIV infection drives proinflammatory adipocyte differentiation in an in vitro model and reveals a new inflammatory pathway
Source: Front Cell Infect Microbiol. 2025 Jul 17;15:1627963. doi: 10.3389/fcimb.2025.1627963 (PMC12310648; doi:10.3389/fcimb.2025.1627963)
Supplement: Supplementary file 1 [file Presentation1.zip › Supplementary Figure 1 legend.docx]

Supplementary Material

**Supplementary Figure 1.** Effect of HIV infection on the expression of *PPAR-γ* (A), *C/EBPα* (B), *C/EBPβ* (C), *HSL* (D), *LPL* (E), *ATGL* (F), *DGAT1* (G), *DGAT2* (H), *SREBP1* (I), *SREBP2* (J), *ADIPOQ* (K) and *LEPTIN* (L) as determined by RT-qPCR at days 1, 3, 7, and 10 post-differentiation. The values are expressed as 2^(−ΔCt)^.
