# Supplementary figures and images for: HIV infection drives proinflammatory adipocyte differentiation in an in vitro model and reveals a new inflammatory pathway
Source: Front Cell Infect Microbiol. 2025 Jul 17;15:1627963. doi: 10.3389/fcimb.2025.1627963 (PMC12310648; doi:10.3389/fcimb.2025.1627963)

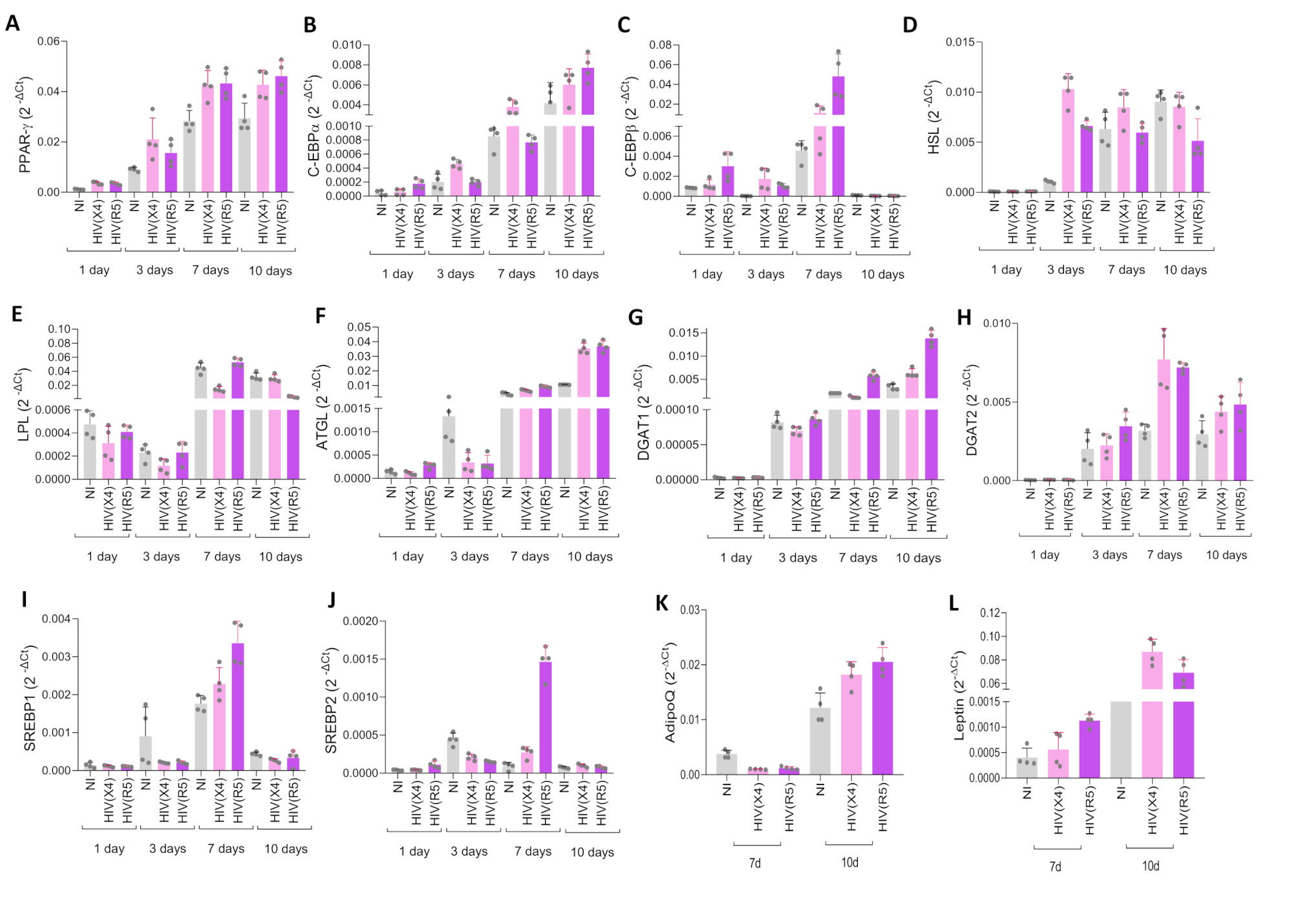

Supplement: Supplementary file 1 [file Presentation1.zip › Suplem Figure 1.tiff]
